# Seasonal and Geographic Variation in Peptic Ulcer Disease and Associated Complications in the United States of America

**DOI:** 10.34172/jrhs.2023.130

**Published:** 2023-12-29

**Authors:** Kausthubha Yaratha, Lindsay Talemal, Brian V. Monahan, Daohai Yu, Xiaoning Lu, Juan Lucas Poggio

**Affiliations:** ^1^Lewis Katz School of Medicine, Temple University, Philadelphia, PA, USA; ^2^Department of Surgery, Temple University Hospital, Philadelphia, PA, USA; ^3^Center for Biostatistics and Epidemiology, Department of Biomedical Education and Data Science, Lewis Katz School of Medicine, Temple University, Philadelphia, PA, USA

**Keywords:** Peptic ulcer disease, Hemorrhage, Perforation, Seasonality, Geographic variation

## Abstract

**Background:** Hospitalization for peptic ulcer disease (PUD) has been described outside of North America as peaking in the fall and winter. However, no recent literature has so far investigated the seasonal fluctuations and complications of PUD in the USA.

**Study Design:** Cross-sectional population database review.

**Methods:** Patients with a diagnosis of either acute gastric or acute duodenal ulcers from January 1, 2015, through December 31, 2017, were identified in the Healthcare Cost and Utilization Project’s National Inpatient Sample. The proportion of admissions with either hemorrhage or perforation was determined for each season and further subdivided into geographic regions.

**Results:** Of 18829 hospitalizations for PUD, admissions were the highest in the fall (25.9%) while being the lowest in the summer (23.9%). Complications, hemorrhage or perforation, were the highest and the lowest in the fall and spring, respectively (75.7% vs. 73.6%; *P*=0.060 for comparing all 4 seasons). Geographically, the West had the highest rate of peptic ulcer hemorrhage (64.5%, *P*=0.004), while the northeast had the highest rate of perforation (14.3%, *P*=0.003). Hemorrhage was more common in males, those who used aspirin, nonsteroidal anti-inflammatory drugs, or anticoagulants, and diabetics (*P*<0.05). Perforation was less common in males, those with diabetes, obesity, or hypertension (HTN), or those using aspirin or anticoagulants (*P*<0.05). *Helicobacter pylori* infection was more associated with perforation in the fall and winter months.

**Conclusion:** Seasonal and regional trends in hospitalizations due to PUD may help identify modifiable risk factors, which can improve diagnostic and treatment outcomes for patients by allowing for more targeted identification of vulnerable populations.

## Background

 Peptic ulcer disease (PUD) and duodenal ulcer disease are characterized by a disruption to the mucosal lining of the stomach or the duodenum. PUD has a lifetime prevalence of approximately 10% in the general population and an incidence of 0.1–0.2% per year.^1,2^ These ulcers can be complicated by hemorrhage in 2–3% of cases per year, and acute mortality may be as high as 12%.^3,4^ However, the incidence and complications of PUD may not be equally distributed throughout the calendar year. Evidence from outside North America shows that PUD, hemorrhage, and perforation peak in the fall and winter.^5,6^ These fluctuations may be caused by factors including seasonal variations in *Helicobacter pylori *infections—a known risk factor for PUD—and gastric mucosa changes related to weather and diet.^7,8^ Many previous studies on this subject focused on smaller countries or regions.

 A broader study of the United States—a country with varied climates and common medical co-morbidities, such as obesity and diabetes—has not been conducted. The study has focused on investigating the seasonal and regional variations of PUD admissions in the United States during the study period. The study will also evaluate whether certain co-morbid conditions are correlated with increased PUD admissions across seasons and regions. Finally, the study will examine whether complicated PUD—cases with hemorrhage or perforation—has seasonal or regional variability.

## Methods

 Data were obtained from the Healthcare Cost and Utilization Project’s National Inpatient Sample (NIS). NIS is the largest publicly available all-payer inpatient database that provides a vast sample of deidentified hospital discharges from 49 states.^9^ The NIS contains admission month, geographic region, discharge diagnoses, procedure codes, and patient characteristics and comorbidities.

 This study evaluated admissions from the three years between January 1, 2015, and December 31, 2017. NIS partitions the United States into the geographic regions of Northeast (New England and the Middle Atlantic), Southeast (South Atlantic, East South Central, and West South Central), Midwest (West North Central and East North Central), and West (Pacific and Mountain). The authors defined seasons using the northern hemisphere meteorological definition, including spring (from March 1 to May 31), summer (from June 1 to August 31), fall (from September 1 to November 30), and winter (from December 1 to February 28).

**Figure 1:**
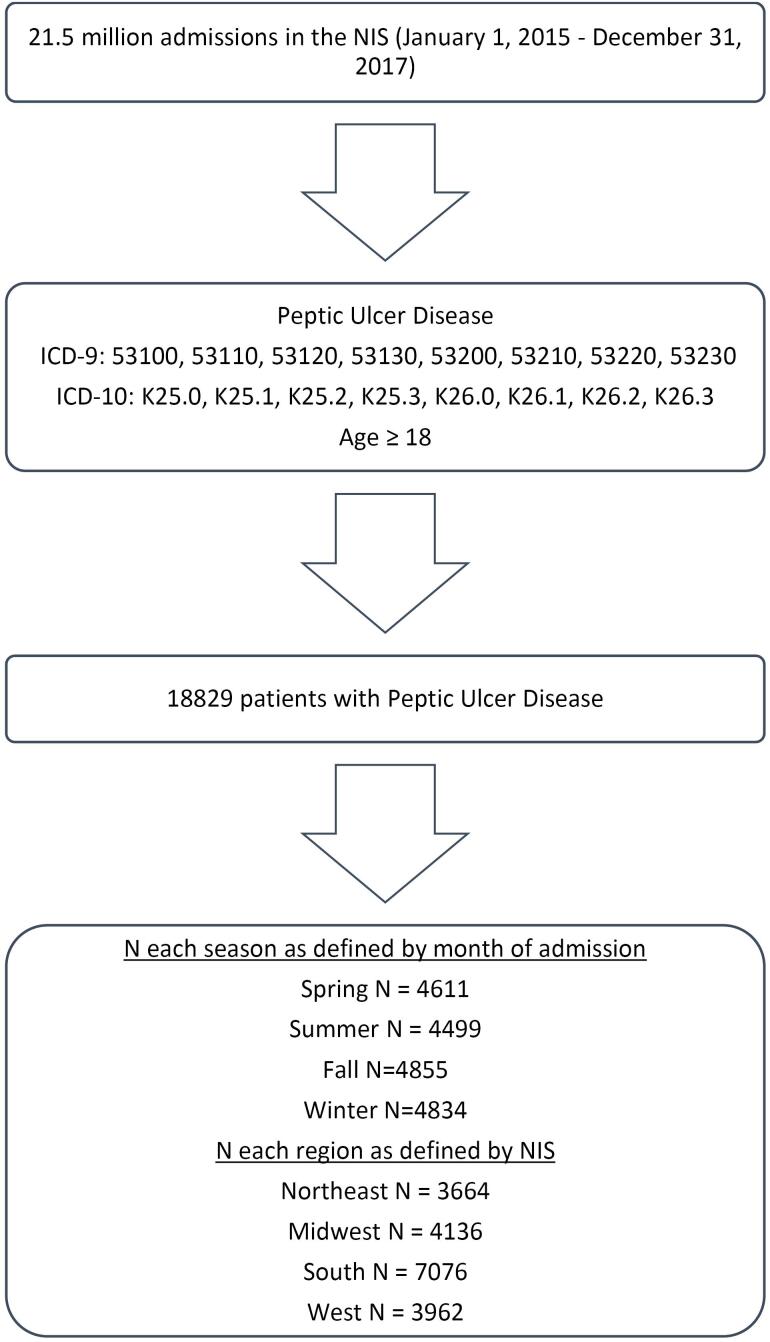
Stepwise data collection

 Both the Ninth and Tenth Editions of the International Classification of Disease, Clinical Modification (ICD-9-CM and ICD-10-CM) codes were used to identify adult (age > 18) hospitalizations with a discharge diagnosis of either acute gastric or acute duodenal ulcers with and without hemorrhage or perforation. The stepwise fashion of data collection is depicted in Figure 1. Patient characteristics and co-morbidities were also collected.

**Figure 2:**
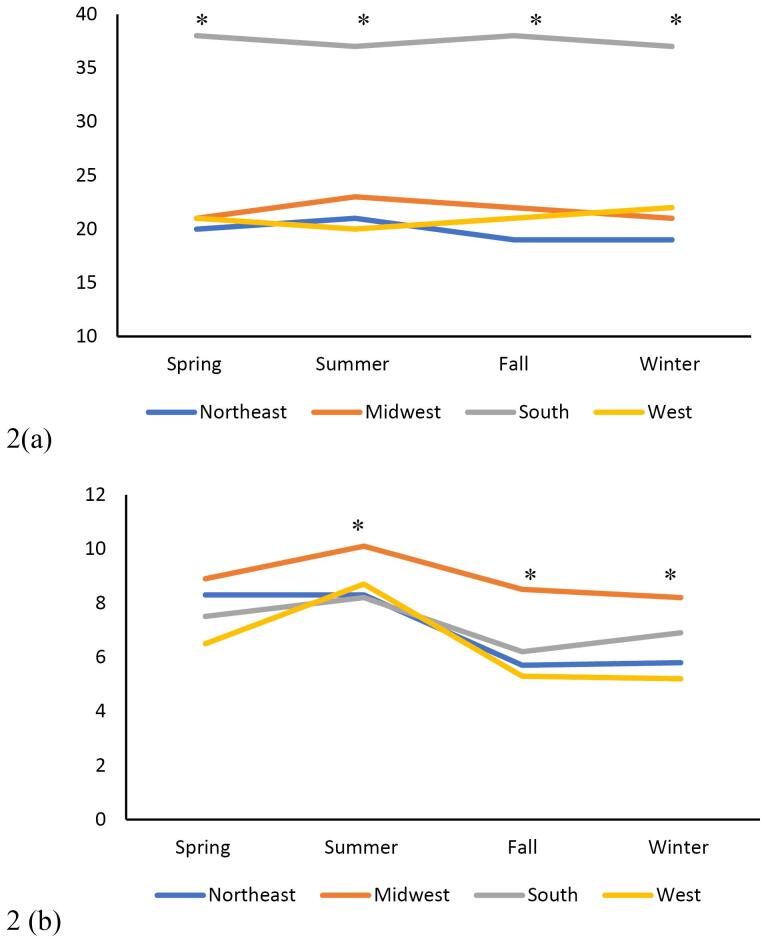
(a) Seasonal overall admission rate by region for peptic ulcer disease in the USA 1/1/15–12/31/17 (* = *P*< 0.05) and (b) Seasonal overall prevalence of patients with BMI > 30 by region for patients admitted with peptic ulcer disease in USA 1/1/15–12/31/17 (* *P*< 0.05). *Note*. BMI, body mass index

**Figure 3:**
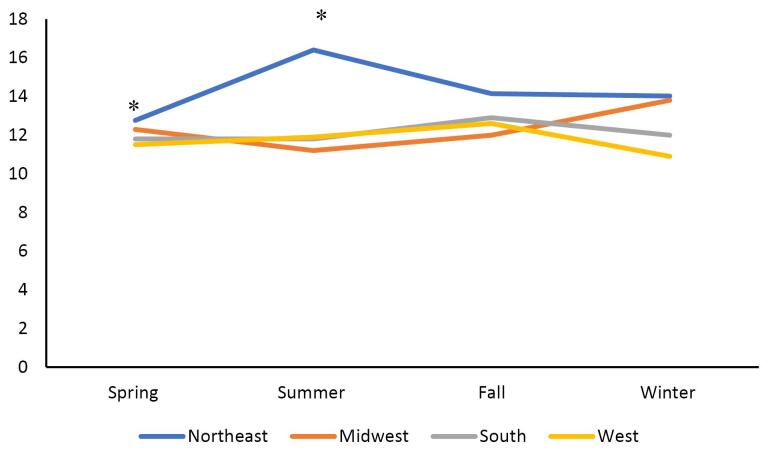
Seasonal overall prevalence of patients With PUD and perforation by region for patients admitted with PUD in USA 1/1/15–12/31/17 (* *P*< 0.05). *Note*. PUD, peptic ulcer disease

**Figure 4:**
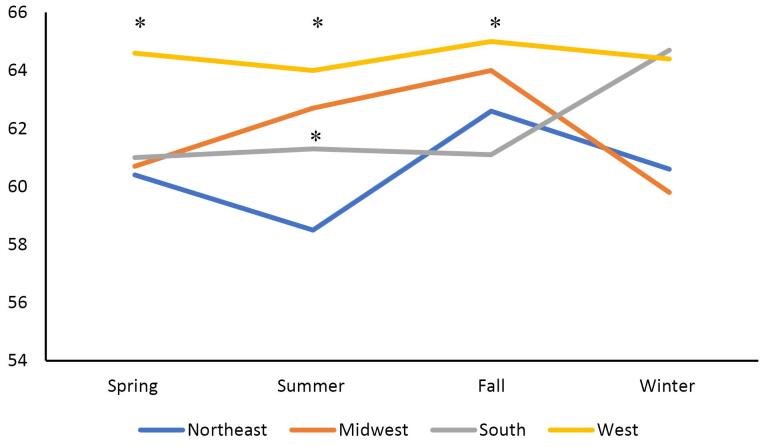
Seasonal overall prevalence of patients with PUD and hemorrhage by region for patients admitted with PUD in USA 1/1/15–12/31/17 (* *P*< 0.05). *Note*.PUD, peptic ulcer disease

**Figure 5:**
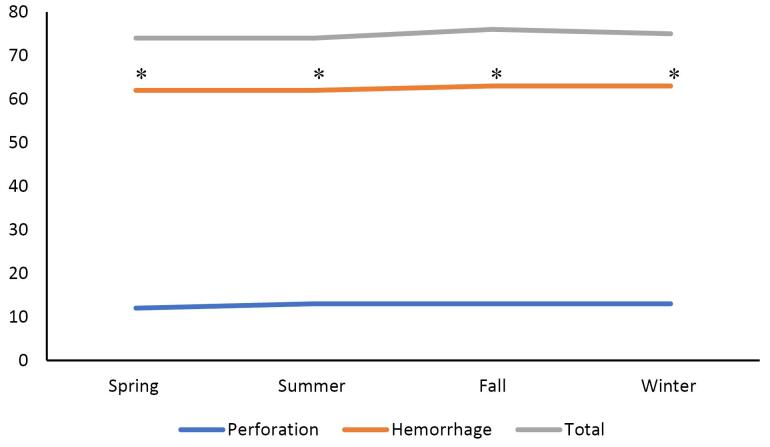
Seasonal overall prevalence of patients with PUD complications (hemorrhage/perforation) for patients admitted with PUD in USA 1/1/15–12/31/17 (* *P*< 0.05). *Note*.PUD, Peptic ulcer disease. Total values were analyzed across the seasons, and *P* value was 0.06. Within every season, the increased prevalence of hemorrhage was statistically significant

 The primary outcome analyzed by this study was the number of patients hospitalized with PUD throughout the spring, summer, fall, and winter, as well as the regions of the Northeast, South, Midwest, and West. Statistical analysis was conducted using the Kruskal-Wallis test for continuous variables and the Chi-square test for categorical variables. Patient demographics and co-morbid conditions, including nonsteroidal anti-inflammatory drug (NSAID) use, hypertension (HTN), obesity (body mass index [BMI] > 30), diabetes, anticoagulant use, hemorrhage, and perforation, were collected and analyzed based on regions and seasons (Figures 2–5). Univariate analysis on the incidence of perforation and hemorrhage by co-morbid conditions was performed as well. This was analyzed using Fisher’s exact test and chi-square tests. Statistical analysis was conducted using SAS, and figures were created using Microsoft Excel.

 Next, multiple regression modeling was created for two combined cohorts with complicated PUD—cases with hemorrhage and perforation. We first identified variables that were clinically relevant to the model. Variables used in modelling perforation were region, season, race, age, diabetes mellitus, *H. pylori* infection, NSAID use, aspirin use, and anticoagulant use. Variables employed in modelling hemorrhage were region, season, gender, race, age, alcohol use, NSAID use, aspirin use, and anticoagulant use. Variables with zero effect—identified by performing joint tests and type 3 analysis of effect tests—were removed from the model, excluding season and region, since these were initial values of interest. For the variables with non-zero effects, we calculated simple differences of variables with the least mean squares, which were adjusted for multiple comparisons using Tukey-Kramer. These provided effect sizes with odds ratios (OR) and confidence intervals (CI).

 Considering that this study utilized and analyzed publicly available, deidentified data, it was deemed exempt from the Institutional Review Board.

## Results

 During the analyzed three-year study period, there were 18 829 hospitalization cases for PUD. By region, the south had the most admissions across all seasons (Figure 2a, *P* < 0.05). There was no statistically significant seasonal variation in PUD admissions. We found a minimal statistical association between PUD and diabetes, HTN, NSAID use, and anticoagulant use, but these associations were likely clinically irrelevant. Patients in the Midwest with PUD and a BMI > 30 were more likely to be hospitalized during the summer, fall, and winter months (*P*< 0.05, Figure 2b).

 The Northeast had the highest rate of PUD and perforation in the summer and fall (14.3%, *P* = 0.003, Figure 3), while the West had the highest rate of peptic ulcer hemorrhage in the spring, summer, and fall (64.5%, *P*= 0.004, Figure 4). Complications—defined by hemorrhage or perforation—were the highest in the fall (75.7%) while being the lowest in the spring (73.6%, *P*= 0.060) for comparing all 4 seasons (Figure 5). Of all complications, hemorrhage was more common across all seasons (*P*< 0.05, Figure 5).

 Across all seasons, there were 18% more cases of hemorrhage in the western region of the US when compared with the Northeast (*P*= 0.004). By season, the winter season showed the greatest regional variability in hemorrhage, with the western region of the US, having 18% more cases when compared to the Northeast. Across all seasons and regions, males were 34% more likely to have hemorrhage than females. Compared to patients less than 60 years old, the risk of hemorrhage was 57% and 86% greater among patients 61–79 years old and patients over 79 years old, respectively (*P*< 0.001). NSAID use was associated with a 37% increase in the risk of hemorrhage (*P*< 0.001). Aspirin use was related to a 76% increase in the risk of hemorrhage (*P*< 0.001). Anticoagulant use was associated with a two-fold increase in the risk of hemorrhage (OR = 2.04, 95% CI: 1.82, 2.30, *P*< 0.001).

 Across all seasons, there were 26% more cases of perforation in the Northeast region of the US when compared with the West (*P*= 0.003). However, during the summer season, the Northeast had 46% more cases than the West (*P*= 0.002). Across all seasons and regions, males were 19% more likely to have hemorrhage than females (*P*< 0.001). Patients less than 60 years old were nearly twice more likely to have perforation than patients over 79 years old (OR = 2.01, 95% CI: 1.78, 2.27, *P* < 0.001). Interestingly, the absence of aspirin and anticoagulation were both independently associated with lower odds of perforation, respectively, with OR = 2.21 (95% CI: 1.89, 2.58) and OR = 2.53 (95% CI: 2.04, 3.14, *P*< 0.001). Interestingly, HTN and obesity were protective against perforation with OR = 0.82 (95% CI: 0.75, 0.89) and OR = 0.84 (95% CI: 0.70, 0.99), respectively (*P*< 0.001, *P*= 0.043).

## Discussion

 The data in this study suggested that there is regional, but not seasonal, variation in the incidence of PUD and associated complications in the United States. This regional difference was predominantly in the south (Figure 2a). There were some trends in regional and seasonal variability in PUD in patients with co-morbid conditions, though these trends were inconsistent. The only statistically significant seasonal variation was the higher rates of PUD in patients with BMI > 30 in the Midwest during summer, fall, and winter (Figure 2b). Hemorrhage was by far the most common complication. The western region’s higher rates of hemorrhage in the spring, summer, and fall, and the northeast region’s higher rates of perforation in the summer were both statistically significant (Figures 4–5). The authors suspect hemorrhage is observed more often due to patients presenting to the healthcare system after hemorrhage and before the occurrence of perforation.

 While not statistically significant, there was a slight increase in the rate of complications in the fall and winter at 75.7% compared with the spring at 73.6% (*P*= 0.060, Figure 5). Worsening outcomes for PUD as the weather cools were also found in foreign studies.^10-12^ Our data support previous studies that show the seasonal variance of *H. pylori* being more prevalent in the colder months. While not observing seasonal variation, PUD with perforation was more associated with *H. pylori* in the fall and winter.^5,7,10^ Many hypotheses have been proposed regarding the etiology of these observations, including gastric mucosal changes, *H. pylori* prevalence, and dietary changes that occur during colder months.^5–7^

 Our data indicated regional differences in the prevalence of PUD. Most notably, people who live in southern states were at higher risk of being admitted for PUD (Figure 2a). This higher risk may be related to people who live in the south, having higher rates of obesity and medical co-morbidities such as diabetes and HTN.^13-15^ Obesity has been implicated as an independent risk factor for PUD.^16^ In a Danish population-based cohort study, diabetes has been shown to increase the risk of short-term mortality in patients with complicated PUD.^17^ Based on our findings, hospital systems in the south can expect to improve gastrointestinal outcomes for their patients by targeting underlying risk factors such as obesity and diabetes.

 Of note, the findings of this study demonstrated that across all seasons and regions, diabetes, coagulopathy, HTN, aspirin use, and anticoagulant use were risk factors for peptic ulcer hemorrhage but were protective factors for perforation. The authors hypothesize that risk factors for hemorrhage are protective against perforation because patients presenting with hemorrhage engage the health system earlier, allowing for earlier treatment and therefore reducing progression to perforation.

 Additionally, female patients were more likely to present with PUD and perforation across most of the studied regions when controlled for all other variables. This is concerning because many studies document differential health care for female patients.^18,19^ Expanding access to care for female patients and recognizing early signs of PUD before progression to perforation would likely be beneficial. Finally, patients who identified as neither black, white, nor Hispanic were more likely to present with hemorrhage and PUD in the western states. This provides an opportunity for outreach to these patients to help mitigate risk factors that may mitigate complicated PUD.

 This study offers a pattern of geographic variation in PUD in the US. While there were some co-morbidity seasonal and regional trends observed in patients admitted with PUD, it is unclear if these are clinically relevant. Additionally, hemorrhage was by far the most common complication of PUD in the United States over the study period. Understanding and appreciating these trends can help physicians and hospitals be proactive. Physicians can counsel their patients on the seasonal and regional risk factors of PUD and associated complications. Additionally, hospital administrators can use this information as they plan staff and resource allocation throughout the year.

 The limitations of the NIS database must be noted. The NIS database is admission-specific, thus oversampling is possible if a patient was admitted multiple times for the same reason. The NIS also have omitted unbilled co-morbid conditions such as obesity. The data do not account for variations in climate within geographic regions, such as the difference between Colorado and California, which are both in the “West” category. In addition, as a database of millions of hospital admissions, human error in coding is possible. While the data are statistically significant, the absolute differences between seasons and geographic regions were modest. Finally, as an observational retrospective study, this study can only report trends and cannot establish causation.

**Highlights**

- Admissions for peptic ulcer disease peak in the fall in the US.
- Peptic ulcer hemorrhage admissions were more likely to occur in the western region of the US.
- Peptic ulcer perforation admissions were more likely to occur in the northeast region of the US.

## Conclusion

 The data revealed significant regional variations in hospital admissions for PUD, with the southern region of the United States being the most common. Seasonal and regional trends in hospitalizations due to PUD may help identify modifiable risk factors, which can improve diagnostic and treatment outcomes for patients by allowing for more targeted identification of vulnerable patients. Patients with risk factors, including HTN, diabetes, obesity, and anticoagulants, had a higher prevalence of hemorrhage but a lower prevalence of perforation. Of all complications, hemorrhage was by far the most common.

## Acknowledgements

 The authors would like to thank the Temple University Hospital Department of Surgery for research support and the Temple University Lewis Katz School of Medicine for their research support.

## Authors’ Contribution

**Conceptualization:** Kausthubha Yaratha, Lindsay Talemal, Juan Lucas Poggio.

**Data curation:** Kausthubha Yaratha, Lindsay Talemal, Brian V. Monahan, Daohai Yu, Xiaoning Lu, Juan Lucas Poggio.

**Formal analysis:** Daohai Yu, Xiaoning Lu.

**Funding acquisition:** Juan Lucas Poggio.

**Investigation: **Kausthubha Yaratha, Lindsay Talemal, Juan Lucas Poggio.

**Methodology:** Kausthubha Yaratha, Lindsay Talemal, Juan Lucas Poggio.

**Project administration:** Juan Lucas Poggio.

**Resources:** Juan Lucas Poggio.

**Software:** Daohai Yu, Xiaoning Lu.

**Supervision:** Juan Lucas Poggio.

**Validation:** Juan Lucas Poggio.

**Visualization:** Kausthubha Yaratha, Lindsay Talemal, Brian V. Monahan, Juan Lucas Poggio.

**Writing–original draft: **Kausthubha Yaratha, Lindsay Talemal, Brian V. Monahan, Juan Lucas Poggio.

**Writing–review & editing:** Kausthubha Yaratha.

## Competing Interests

 The authors have no financial disclosure.

## Funding

 This research received no outside funding.
